# Supplementary material for: Measurement properties of pain scoring instruments in farm animals: A systematic review using the COSMIN checklist
Source: PLoS One. 2023 Jan 20;18(1):e0280830. doi: 10.1371/journal.pone.0280830 (PMC9858734; doi:10.1371/journal.pone.0280830)
Supplement: S1 Table — (DOCX) [file pone.0280830.s001.docx]

S1 Supplementary Methods

Table S1. Detailed criteria used for assessing methodological quality of each included study.

| **Component of the scale** | **Category** | **Criteria** | **Scoring guidelines** |
| --- | --- | --- | --- |
| **Scale Development** | **1a. General design requirements and development** | 1. Is a clear description provided of the construct to be measured? | V - Construct clearly described (type of pain, etc.)  I - Not clearly described |
|  |  | 2. Is the origin of the construct clear: was a theory, conceptual framework or disease model used or clear rationale provided to define the construct to be measured? | V - Origin of the construct clear  D - Origin not clear |
|  |  | 3. Is a clear description provided of the target population and context for which the scale was developed? | V - Target population and context clearly described  I - Not clearly described |
|  |  | 4. Was the scale development study performed in a sample representing the target population? | V - Study performed in a sample representing the target population  A - Assumable that the study was performed in a sample representing the target population, but not clearly described  D - Doubtful whether the study was performed in a sample representing the target population  I - Study not performed in a sample representing the target population |
|  |  | 5. Was an appropriate method used to identify relevant items or AU for a new scale? | V - Method based on ethogram or underlying anatomy or systematic coding of action of muscles available and used for reference (i.e. FACS)  A - Systematic comparison of images (using qualitative or quantitative criteria) for identification of items or AU or based in the literature  D - Not clear  I - Items / AU listed based on other species |
|  |  | 6. Was a skilled observer or group of observers (experts in the field) used to define the items? | V - More than one skilled observer and they did not take part in subsequent validation of the scale  A - One skilled observer was involved in the development and did not take part in subsequent validation or justification provided or more than one skilled observer participates both scale development and validation  D - Not clear if the same observer(s) was(were) involved in the development and validation of the scale  I - Observers were not skilled or experts in the field |
|  |  | 7. Were the animals undisturbed during evaluation (or was the effect of handling/observer accounted)? | V - (Animals undisturbed) observer not present  A - Effect of handling accounted or observer effect accounted  D - Unclear or handling/observer effect acknowledged as a limitation or attempted to minimize stress  I - Clear signal of disturbance |
|  | **1b. Content validity and comprehensibility** | 1. Was the content validity stablished? | V - Content validity index calculated based on the expert opinion  A - Based on expert opinion without an index  D - Not clear  I - Not performed  N - Not applicable or grimace scales |
|  |  | 2. Was an appropriate method used to ask professionals whether each item is relevant for the construct of interest? | V - Content validity index calculated based on the expert opinion  A - Based on expert opinion without an index  D - Not clear  I - Not performed  N - Not applicable or grimace scales |
|  |  | 3. Was an appropriate method used to ask professionals whether each item is clear for the construct of interest? | V - Content validity index calculated based on the expert opinion  A - Based on expert opinion without an index  D - Not clear  I - Not performed  N - Not applicable or grimace scales |
|  |  | 4. Does the scale include descriptors of both normal and pain related behaviors? | V - Animals undisturbed  A - Effect of handling accounted  D - Handling acknowledged as a limitation  I - Effect of handling/physical restraint not accounted |
|  |  | 5. Was the comprehensibility evaluated by the end-user? | V - Performed  D - Unclear  N - Not applicable or grimace scales |
|  |  | 6. Was an appropriate method use to assess the comprehensibility - regarding to instructions, items, and response options? | V - A systematical approach used  A - Defining during interview/discussion  D - Unclear  N - Not applicable or grimace scales |
| **Measurement Properties** | **2a. Internal consistency** | 1. Was the internal consistency calculated and reported? | V - Internal consistency was calculated and reported (Cronbach's alpha or omega or KR-20 calculated) or Linear discriminant analysis (LDA)  A - Only item-total (or inter-items) correlations calculated  D - Unclear  N - Not calculated / Not applicable |
|  |  | 2. Were there any other important flaws? | V - No other important methodological flaws  A - Other minor methodological flaws  D - Unclear  I - Other important methodological flaws |
|  | **2b. Reliability** | 1. Was inter-rater reliability reported? | V - Yes; inter-rater reliability calculated and reported  D - Inter-rater reliability not clearly reported and/or Unclear  N - Not reported / Not applicable |
|  |  | 1.1. Was the number of raters appropriate for inter-rater reliability testing? | V - Number of raters appropriate (justification provided) equal or greater than three  D - Number was not clearly stated  I - Number of raters not appropriate (two) |
|  |  | 1.2 Was the statistical method for calculating inter-rater reliability appropriate? | V - ICC calculated and model or formula is described, or Weighted kappa calculated (well described, ICC estimated, and 95% CI calculated based on single measures using a two-way random effects model for absolute agreement or consistency) or Kendal's concordance coefficient  A - ICC calculated but model or formula not described or not optimal  D - Unweighted Kappa calculated instead of Weighted kappa for ordinal scores  I - No ICC or Kappa/Weighted kappa reported, or correlation to evaluate |
|  |  | 2. Was intra-rater reliability reported? | V - Yes intra-rater reliability calculated and reported, and Evidence provided, that patients were stable  A - Intra-rater reliability calculated and Assumable that patients were stable  D - Intra-rater not clearly reported and/or Unclear if patients were stable  I - Evidence that patients were not stable  N - Not reported / Not applicable |
|  |  | 2.1 Was the time interval appropriate for intra-rater reliability testing? | V - Time interval appropriate (at least a month)  D - Whether time interval was appropriate or time interval was not stated  I - Time interval not appropriate  N - Not reported / Not applicable |
|  |  | 2.2 Were the test conditions similar for the measurements? e.g. type of administration, environment, instructions | V - Test conditions were similar (evidence provided) using video/image  A - Assumable that test conditions were similar  D - Unclear if test conditions were similar  I - Test conditions were not similar (e.g. training between sections)  N - Not reported / Not applicable |
|  |  | 2.3 Was the statistical method for calculating intra-rater reliability appropriate? | V - ICC calculated and model or formula is described, or Weighted kappa calculated (well described, ICC estimated, and 95% CI calculated based on single measures using a two-way random effects model for absolute agreement or consistency)  A - ICC calculated but model or formula not described or not optimal  D - Unweighted Kappa calculated instead of Weighted kappa for ordinal scores  I - No ICC or Kappa/Weighted kappa reported, or correlation to evaluate |
|  |  | 3. Were there any other important flaws? | V - No other important methodological flaws  A or D - Other minor methodological flaws - not clear if observer was aware of previous scores for same animal  I - Other important methodological flaws |
|  | **2c. Measurement error** | 1. Were sensitivity, specificity and/or accuracy determined? | V - Sensitivity, specificity, and accuracy (% or AUC) calculated for the scale's score  A - Possible to calculated from the data presented*  D - Unclear  I - Sensitivity, specificity and accuracy calculated based on a dichotomous global evaluation based on observer opinion (presence or absence of pain)  N - Not calculated or presented |
|  |  | 2. Were there any other important flaws? | V - No other important methodological flaws  A or D - Other minor methodological flaws (if only presential evaluation and/or not blinded)  I - Other important methodological flaws |
|  | **2d. Criterion validity** | 1. Was criterion validity reported? | V - Criterion validity was calculated and reported  D - Unclear  N - Not calculated / Not applicable |
|  |  | 2. Is it clear what the gold standard or other method measure(s)? | V - Construct is clear  D - Unable to determine  I - Construct is not clear |
|  |  | 3. Were the measurement properties of the gold standard or other validated method adequate? | V - Sufficient evidence of the measurement properties of the gold standard or other method provided (reference included)  A - Partial evidence of the measurement properties of the gold standard or other method provided  D - No information on the measurement properties of the gold standard or other method  I - Evidence for insufficient measurement properties of the gold standard or other method |
|  |  | 4. Was the statistical method appropriate for the hypotheses to be tested? | V - Statistical method was appropriate (correlations or area under the ROC curve calculated)  A - Assumable that statistical method was appropriate  D - Statistical method applied not optimal or not clearly reported  I - Statistical method applied not appropriate |
|  |  | 5. Were there any other important flaws? | V - No other important methodological flaws  A or D - Other minor methodological flaws  I - Other important methodological flaws |
|  | **2e. Construct validity: Comparison between subgroups (discrimination between painful and pain-free animals)** | 1. Was construct validity reported? | V - Construct validity was reported  D - Unclear  I - Construct validity not reported (in the first study of the scale)  N - Not reported in subsequent studies |
|  |  | 2. Was an adequate description provided of important characteristics of the subgroups? | V - Adequate description of the important characteristics of the subgroups (minimum age, sex)  A - Adequate description of most of the important characteristics of the subgroups  D - Poor or no description of the important characteristics of the subgroups |
|  |  | 3. Was the statistical method appropriate for the hypotheses to be tested? | V - Statistical method was appropriate  A - Assumable that statistical method was appropriate  D - Statistical method applied not optimal or not clear  I - Statistical method applied not appropriate |
|  |  | 4. Were there any other important flaws? | V - No other important methodological flaws  A or D - Other minor methodological flaws  I - Other important methodological flaws |
|  | **2f. Responsiveness (discrimination between before and after analgesic intervention)** | 1. Was responsiveness reported? | V - Responsiveness was calculated and reported  D - Unclear  N - Not calculated / Not applicable |
|  |  | 2. Was an adequate description provided of the intervention given? | V - Adequate description of the intervention (i.e. dose, route of administration, etc.) and appropriate interval (before-after) considered  A - Description of the intervention not optimal (i.e. dose, route of administration, etc.) and/or interval (before-after) not optimal  D - Poor description of intervention and/or interval (before-after)  I - No description of the intervention and/or interval (before-after) not appropriate |
|  |  | 3. Was the statistical method appropriate for the hypotheses to be tested? | V - Statistical method was appropriate  A - Assumable that statistical method was appropriate  D - Statistical method applied not optimal  I - Statistical method applied not appropriate |
|  |  | 4. Were there any other important flaws? | V - No other important methodological flaws  A or D - Other minor methodological flaws  I - Other important methodological flaws |
|  | **2g. Cross-cultural validity** | 1. Were translation and back translation performed? | V - Different observers fluent in both languages, included one native speaker of the target language  A - Same individual or individual not native speaker, at least one month later  D - Unclear or not clearly reported  I - Same observer at once  N - Not applicable for original studies |
|  |  | 2. Were the samples similar for relevant characteristics? | V - Evidence provided that samples were similar  A - Stated or assumable that samples were similar  D - Unclear  I - Samples not similar  N - Not applicable for original studies |
|  |  | 3. Were there any other important flaws? | V - No other important methodological flaws  A or D - Other minor methodological flaws (different methods of analysis from the original study)  I - Other important methodological flaws (major bias or confounding)  N - Not applicable for original studies |

AU: action units. FACS: facial action coding system. AUC: area under the curve. ICC: intraclass correlation coefficient. KR-20: Kuder–Richardson Formula 20. Each criterion was independently scored by two individuals as ‘V’ (very good), ‘A’ (adequate), ‘D’ (doubtful), ‘I’ (inadequate) or ‘N’ (not applicable). *If sensitivity and specificity were presented, accuracy can be calculated.
