# Supplementary material for: Measurement properties of pain scoring instruments in farm animals: A systematic review using the COSMIN checklist
Source: PLoS One. 2023 Jan 20;18(1):e0280830. doi: 10.1371/journal.pone.0280830 (PMC9858734; doi:10.1371/journal.pone.0280830)
Supplement: S2 Table — (DOCX) [file pone.0280830.s002.docx]

S2 Supplementary Methods

Table S2. Summary of the population characteristics in the studies included in the systematic review.

| Species / Scale | Ref | Number of animals | Age | Sex (%) | Breed / Strain |
| --- | --- | --- | --- | --- | --- |
| Bovine / UCAPS | [23] | 40 cattle | 1-3 years | M (100%) | Nelore |
|  | [60] | 40 cattle | 1-3 years | M (100%) | Nelore |
| Bovine / PSS | [17] | 345 dairy cattle | NR | F (100%) | NR |
| Bovine / MPSS | [49] | 16 cows | NR | F (100%) | Holstein and Swiss Fleckvieh |
| Bovine / EA | [50] | 8 calves | 200 ± 5 days | M (100%) | Holstein BW |
| Bovine / VPS | [51] | 12 cows | Average 4.8 years | F (100%) | Holstein |
| Bovine / TPS | [51] | 12 cows | Average 4.8 years | F (100%) | Holstein |
| Bovine / CPS | [35] | 139 cows | NR | F (100%) | Danish Holstein and Friesian |
| Bovine / PABFE | [56] | 45 bulls | 20 ± 2 months | M (100%) | Nelore |
| Ovine / SPFES | [13] | 140 sheep | Footrot >1 year  Mastitis NR | Footrot NR  Mastitis F (100%) | Various breeds |
| Ovine / PSS-VADS | [52] | 10 sheep | 3-4 months | NR | Dorset crossbred |
| Ovine / BAS | [53] | 24 sheep | 1 year | M (100%) | Texel |
| Ovine / SGS | [24] | 14 sheep | 3-4 years | F (100%) | Blackface sheep |
| Ovine / lgs | [57] | 18 lambs | 5-6 weeks | F (45%)  M (55%) | Romney cross |
| Ovine / usaps | [26] | 48 sheep | 3.5 ± 1.8 years | F (100%) | Bergamacia,  Lacaune and Dorper |
| Porcine / UPAPS | [22] | 45 pigs | 38 ± 3 days | M (100%) | Landrace, Large White, Duroc, and Hampshire |
| Porcine / PGS-B | [58] | 19 piglets | 5-day-old | M (100%) | Yorkshire |
|  | [63] | 120 piglets | 5-day-old | M (100%) | Yorkshire-Landrace cross Duroc |
|  | [62] | 68 piglets | 5-day-old | M (100%) | Yorkshire-Landrace cross Duroc |
|  | [61] | 10 pigs | 73 ± 10.6 days | M (100%) | Large White cross Duroc |
| Porcine / PGS-A | [14] | 23 piglets | 3-4 days old | F (35%)  M (65%) | Landrace, Large White, and Belgian Landrace |
| Porcine / SFES | [59] | 21 sows | NR | F (100%) | Danbred |
| Porcine / PDD | [54] | 66 pigs | 2-3 months | M (100%) | Large White |
| Porcine / BPSP | [55] | 500 piglets | 7 days old | M (100%) | NR |

Ref: Reference number between brackets. UCAPS: Unesp-Botucatu Unidimensional Composite Pain Scale for assessing postoperative pain in cattle. PSS: Posture Scoring System. MPSS: Multidimensional Pain Scoring System. EA: Escala Composta Análogo-Visual. VPS: Veterinarian Pain Scale. TPS: Technician Pain Scale. CPS: Cow Pain Scale. PABFE: Pain Assessment Based on Facial Expression. SPFES: Sheep Pain Facial Expression Scale. PSS-VADS: Pain Scoring System for Ventricular Assist Devices-Implanted Sheep. BAS: Behavior Assessment Scheme. SGS: Sheep Grimace Scale. LGS: Lamb Grimace Scale. USAPS: Unesp-Botucatu Composite Scale to Assess Acute Postoperative Abdominal Pain in Sheep. UPAPS: Unesp-Botucatu Pig Composite Pain Scale. PGS-B: Piglet Grimace Scale-b. PGS-A: Piglet Grimace Scale-a. SFES: Sow Facial Expression Scale. PDD: Perception of Pain, Distress and Discomfort Assessment. BPSP: Behavioral Pain Scale in Piglets. NR: Not reported. Note: Data retrieved from the articles included in this systematic review and reported herein are subject to bias or error attributable to any misinterpretation or unclear reporting
