# Supplementary material for: Measurement properties of pain scoring instruments in farm animals: A systematic review using the COSMIN checklist
Source: PLoS One. 2023 Jan 20;18(1):e0280830. doi: 10.1371/journal.pone.0280830 (PMC9858734; doi:10.1371/journal.pone.0280830)
Supplement: Attachment — Submitted filename: Response to reviewers.docx [file pone.0280830.s005.docx]

Reviewer #2: Thank you for the revised manuscript and clarifying answers in the attached response letter. My comments have been addressed more or less sufficiently apart from the issue of sample sizes.

Although, sample sizes are given in Table S2 and should be covered by your procedure according to Table 1, the issue of small vs. larger sample sizes does play a pivotal role for the specific validity measures of each study in terms of ICC/IOR and accuracy estimates. E.g. for cattle pain scoring instruments sample sizes in the included studies vary from as little as 8 to 345 animals. I think this is worth mentioning in the discussion.

Answer: Thank you for reviewing the manuscript once again. A comment has been added to the discussion: “Additionally, results of reliability testing and other measurement properties could have been influenced by the sample size (i.e. number of animals included) among studies. Indeed, the COSMIN criteria do not take study sample size in consideration during methodological quality assessment”.

Table 6 - Are footnotes for the letters a-j missing?

Answer: Footnotes have been added.
